# The Looming Effects of Estrogen in Covid-19: A Rocky Rollout

**DOI:** 10.3389/fnut.2021.649128

**Published:** 2021-03-18

**Authors:** Hayder M. Al-kuraishy, Ali I. Al-Gareeb, Hani Faidah, Thabat J. Al-Maiahy, Natália Cruz-Martins, Gaber El-Saber Batiha

**Affiliations:** ^1^Department of Clinical Pharmacology and Therapeutic Medicine, College of Medicine, Al-Mustansiriyiah University, Baghdad, Iraq; ^2^Microbiology, Faculty of Medicine, Umm Al Qura University, Mecca, Saudi Arabia; ^3^Department of Gynecology and Obstetrics, College of Medicine, Al-Mustansiriyiah University, Baghdad, Iraq; ^4^Faculty of Medicine, University of Porto, Porto, Portugal; ^5^Institute for Research and Innovation in Health (i3S), University of Porto, Porto, Portugal; ^6^Laboratory of Neuropsychophysiology, Faculty of Psychology and Education Sciences, University of Porto, Porto, Portugal; ^7^Department of Pharmacology and Therapeutics, Faculty of Veterinary Medicine, Damanhour University, Damanhour, Egypt

**Keywords:** Covid-19, estrogen, angiotensin converting enzyme-2, SARS-CoV-2, patients outcomes

## Abstract

In the face of the Covid-19 pandemic, an intensive number of studies have been performed to understand in a deeper way the mechanisms behind better or worse clinical outcomes. Epidemiologically, men subjects are more prone to severe acute respiratory syndrome-coronavirus type 2 (SARS-CoV-2) infections than women, with a similar scenario being also stated to the previous coronavirus diseases, namely, SARS-CoV in 2003 and Middle East Respiratory Syndrome coronavirus diseases (MERS-CoV) in 2012. In addition, and despite that aging is regarded as an independent risk factor for the severe form of the disease, even so, women protection is evident. In this way, it has been expected that sex hormones are the main determinant factors in gender differences, with the immunomodulatory effects of estrogen in different viral infections, chiefly in Covid-19, attracting more attention as it might explain the case-fatality rate and predisposition of men for Covid-19 severity. Here, we aim to provide a mini-review and an overview on the protective effects of estrogen in Covid-19. Different search strategies were performed including Scopus, Web of Science, Medline, Pubmed, and Google Scholar database to find relative studies. Findings of the present study illustrated that women have a powerful immunomodulating effect against Covid-19 through the effect of estrogen. This study illustrates that estrogens have noteworthy anti-inflammatory and immuno-modulatory effects in Covid-19. Also, estrogen hormone reduces SARS-CoV-2 infectivity through modulation of pro-inflammatory signaling pathways. This study highlighted the potential protective effect of estrogen against Covid-19 and recommended for future clinical trial and prospective studies to elucidate and confirm this protective effect.

## Background

Coronavirus disease 19 (Covid-19) is a global pandemic danger caused by the severe acute respiratory syndrome-coronavirus type 2 (SARS-CoV-2). SARS-CoV-2 spike protein binds to angiotensin-converting enzyme 2 (ACE2) receptors (1), which are involved in the viral entry. Such receptors are highly expressed in different tissues, mainly in lung pneumocyte type II cells, with SARS-CoV-2 bindings to ACE2 leading to downregulation of protective ACE2 and induction of hyper-inflammation and oxidative stress, with consequent progress of acute lung injury (ALI) and acute respiratory distress syndrome (ARDS) (2). Also, reduction of ACE2 leads to vasoconstriction, hypertension, coagulopathy, and induction of inflammatory reactions that together increase the risk of ALI and Covid-19 severity (3).

From an epidemiological point of view, and specifically looking at gender differences, men subjects are more prone and vulnerable to SARS-CoV-2 infection than women, about 2.4 times higher due to hormonal differences and expression, as well as distribution of ACE2 (4). Previous coronavirus diseases, including severe acute respiratory syndrome coronavirus (SARS-CoV) in 2003 and the Middle East Respiratory Syndrome coronavirus disease (MERS-CoV) in 2012, showed the same pattern in gender susceptibility. For instance, in SARS-CoV, the case-fatality rate was 22% for men compared to 13% for women, while in MERS-CoV the case-fatality rate was 52% for men compared to 23% for women (5). In SARS-CoV-2, different studies have underlined that females are less prone to infection, contributing for 18% of total Covid-19 cases compared to the affected matched male subjects (6). In United Kingdom, a large-prospective cohort study illustrated that women account for 40% of Covid-19 cases with 20% lower case-fatality rate compared to men (7). However, aging is regarded as an independent risk factor for both genders in the development of severe Covid-19; even so, women protection is still evident (8). In fact, women differ from men in both nutritional requirements and energy consumption based on sex hormone differences. Also, the rate of infection in men compared to women might be due to different other reasons, including the following: men more prevalently work outside the house or work in places that put them to virus exposure; women, due to the low rate of health insurance or other social issues, less frequently go for a test and seek medical attention unless the symptoms are serious. Also, statistical bias in different studies may affect the prevalence and incidence of various infections in regard to gender difference (9). In general, women have a higher immune response against different pathogens due to underlying genetic (two X chromosomes) and hormonal differences (10). The presence of two X chromosomes in women affects the immune system even if one is inactive. The X chromosome acts on various elements of the immune system such as Toll-like receptors (TLRs) and chemokines which can be overexpressed in women and influence the response to viral infections and vaccinations (11). Likewise, the levels of activation of the immune cells affected by sex hormone are higher in women than in males, and this is linked with the stimulation of TLR7 and the production of interferon gamma (INF-γ). Hence, related to the X chromosomes, TLR7 is higher in women than in men and its expression leads to higher immune responses, although these reactions can cause autoimmune phenomena (12). Also, both immunoglobulins and circulating T cells are higher in women compared to men, so that women's immune reactivity is more active against different viral infections, which predispose them for risk of autoimmunity (13). Furthermore, women have an average higher frequency of circulating CD4 T cells than men and clinical studies reveal that men have lower CD3 and CD4 T cell counts, CD4/CD8 T cell ratios, and helper T cell type 1 (Th1) responses than women; thus, cytokine productions in response to infections are enhanced in women compared with men (13).

On the other hand, it has been proposed that pathogen-associated molecular patterns (PAMPs) bind to TLRs. TLR2 and TLR4 which bind bacterial cell wall proteins are more evident in men compared to TLR3, TLR7, and TLR9 which bind viral proteins in women (14). Thus, the production of interferon (mediated by TLR7) and IL-10 (mediated by TLR9) are lower in men during viral infections (15, 16). Also, women tend to activate type 2 immune response, which engaged with the production of anti-inflammatory cytokines, while men tend to activate type 1 immune response, which are involved in the production of pro-inflammatory cytokines (17). Taken together, men subjects have a lower level of regulatory T cells (Treg) during cytomegalovirus infections compared to women, suggesting a propensity for activation of type 1 immune response in men with production of pro-inflammatory cytokines (18, 19). Different studies have documented that sex steroids are the main determinant factors in gender immune differences mainly at the reproductive age (20), with the immunomodulatory effects of estrogen, progesterone, and androgen in different viral infections, chiefly in Covid-19, attracting more attention as it might explain the case-fatality rate and men's predisposition for Covid-19 severity, since serum concentrations of sex hormones change across life span (21). Here, we aim to provide an overview on the gender-specific immunological profile and the protective effects of estrogen in Covid-19.

## Method and Search Strategy

**Figure 1:**
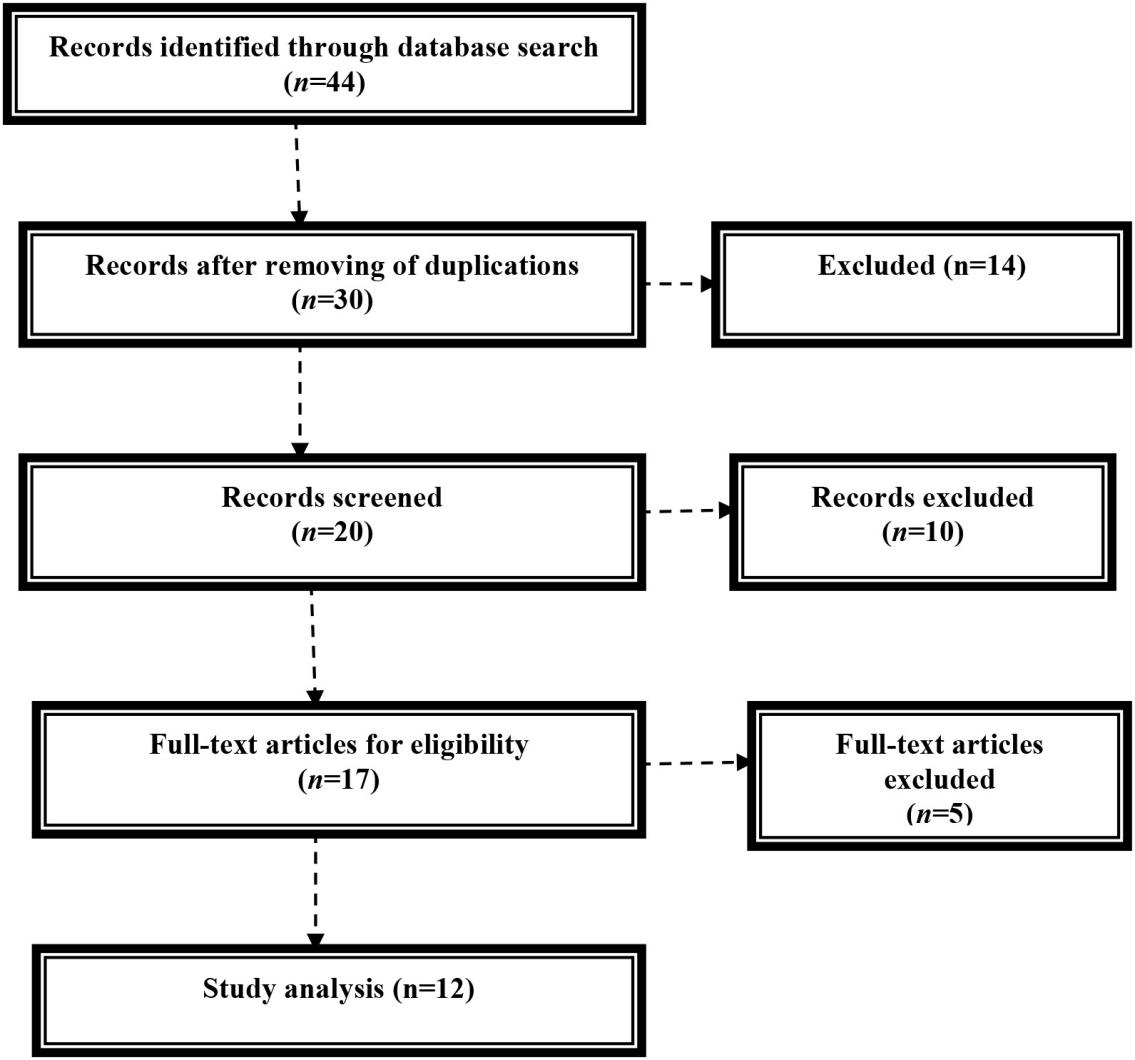
Consort flowchart of the present study.

The literature and study search were performed according to the guideline of systemic review. We search PubMed, Scopus, Web of Science, and Google Scholar database by using sequence keywords including (SARS-CoV-2 OR Covid-19) AND (acute respiratory syndrome OR acute lung injury), (SARS-CoV-2 OR Covid-19) AND (Gender differences OR hormonal effects), (SARS-CoV-2 OR Covid-19) AND (Estrogen OR estrogen agonists), (SARS-CoV-2 OR Covid-19) AND (women OR men), and (SARS-CoV-2 OR Covid-19) AND (immunological differences OR Covid-19 manifestations). The literature search was organized independently by all authors throughout searching the titles and abstracts of regained articles. All of the published and preprinted studies were integrated in this study without limitation of languages. Following the preliminary search and screening, the selected articles were experienced for eligibility and summarized in a mini-review (Figure 1).

## Estrogen and Covid-19

Estradiol (E2) binds to the cytoplasmic estrogenic receptors (ER-α and ER-β) that are expressed on T cells and B cells, respectively (22). Momentarily, E2 activates humoral immunity and antibody production against different viral infections (23). High E2 concentration is correlated with immune-reactivity around ovulation (24). Both hormone replacement therapy after menopause and use of combined contraceptive pills produce a similar potent immune response and protection against viral infections (25). Besides, progesterone and testosterone have immunosuppressive effects on cell-mediated response and innate immunity. However, progesterone inhibits type 1 immune response as well as cytotoxic T and natural kill cells with activation of type 2 immune response, and this might explain the association between the use of progestin-only contraceptive depot and the risk of human immune deficiency virus (HIV) (26). Ruggieri et al. (27) found the association between testosterone use and the risk of viral infections including hepatitis C and B due to suppression of T cells and production of interferon gamma (IFN-γ). E2 has potent anti-inflammatory and immunomodulation effects in both humans and animals. E2 inhibits innate immunity through suppression of immune cell migration, mainly neutrophils and monocytes to the inflamed area. This effect attenuates the development and progression of cytokine storm (CS) via inhibition of pro-inflammatory cytokine release (i.e., IL-1β, IL-6, IL-17, TNF-α, and chemokines) (28). CS is regarded as a major cause for the development of ALI, ARDS, multiorgan failure, and death in Covid-19 (29). In addition, physiological E2 activates the production of anti-inflammatory cytokines (IL-4, IL-10) and promotes immune tolerance through stimulation of CD4 and regulatory T cells (Treg), although low dose of E2 promotes pro-inflammatory activation (30). However, Bommer et al. (31) showed that higher E2 concentration during pregnancy inhibits the anti-inflammatory IL-10 without effect on the pro-inflammatory profile. In Covid-19, a higher circulating Treg and anti-inflammatory cytokines have been linked to a rapid recovery and good clinical outcomes (32). In fact, a case–control study by Neumann et al. (33) proposed that higher IL-10-producing Treg cells are linked to Covid-19 severity. However, high IL-10-producing Treg cells might be a compensatory mechanism to overcome high pro-inflammatory cytokines (34). In addition, E2 inhibits the synthesis and release of IL-6 in different acute and chronic inflammatory disorders, like ALI by inhibiting IL-6 (35). Indeed, IL-6 antagonist tocilizumab is an effective agent in the ARDS management of Covid-19, suggesting the critical role of IL-6 in ALI pathogenesis and Covid-19 severity (36). Experimental studies have shown that lipopolysaccharide (LPS)-induced ALI and high IL-6 levels are higher in ovariectomized female and male mice (37).

Different clinical studies documented the protective role through various pathways. In Italy, the case-fatality rate is higher in men at about 63.9% compared with affected women due to hormonal changes and lifestyle diversity (38). Shabbir et al.'s (39) observational study involved 162,392 Covid-19 patients which showed that 74% of affected patients were men compared to 26% women due to the protective effect of estrogen. Moreover, Garg et al. (40) revealed that the mortality rate was higher in postmenopausal (12.8%) compared with premenopausal women (8.6%), highlighting the protective role of estrogen against Covid-19.

**Figure 2:**
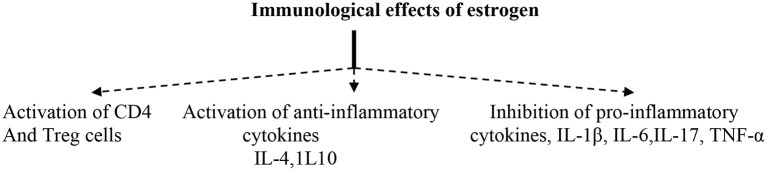
Immunological effects of estrogens.

On the other hand, it has been reported that endogenous E2 leads to significant pulmonary protection against influenza and SARS viruses in previous preclinical studies through anti-inflammatory effects. Also, the use of fulvestrant (ER full antagonist) in women increases the risk of SARS-induced ALI comparable to men, suggesting of a potential lung-protective effect of endogenous estrogen (41). Likewise, selective estrogen receptor modulators (SERMs), like tamoxifen and toremifene, have noteworthy protective effects against ALI in SARS-CoV and MERS-CoV. Also, these drugs have antiviral effects against different coronaviruses through inhibition of viral entry and replication, along with cytopathic effects (42). Specifically, tamoxifen blocks the mitochondrial complex and oxygen consumption of infected cells through activation of AMPK signaling. In addition, endogenous estrogen and SERMs modulate the SARS-CoV-2 binding to the entry point ACE2 (43). Since SARS-CoV has a higher similarity with SARS-CoV-2, thus estrogen may be an effective agent against Covid-19-induced ARDS. Therefore, the net effects of estrogen on the inflammatory profile during SARS-CoV-2 are summarized (Figure 2).

## Estrogen and Renin–Angiotensin System in Covid-19

**Figure 3:**
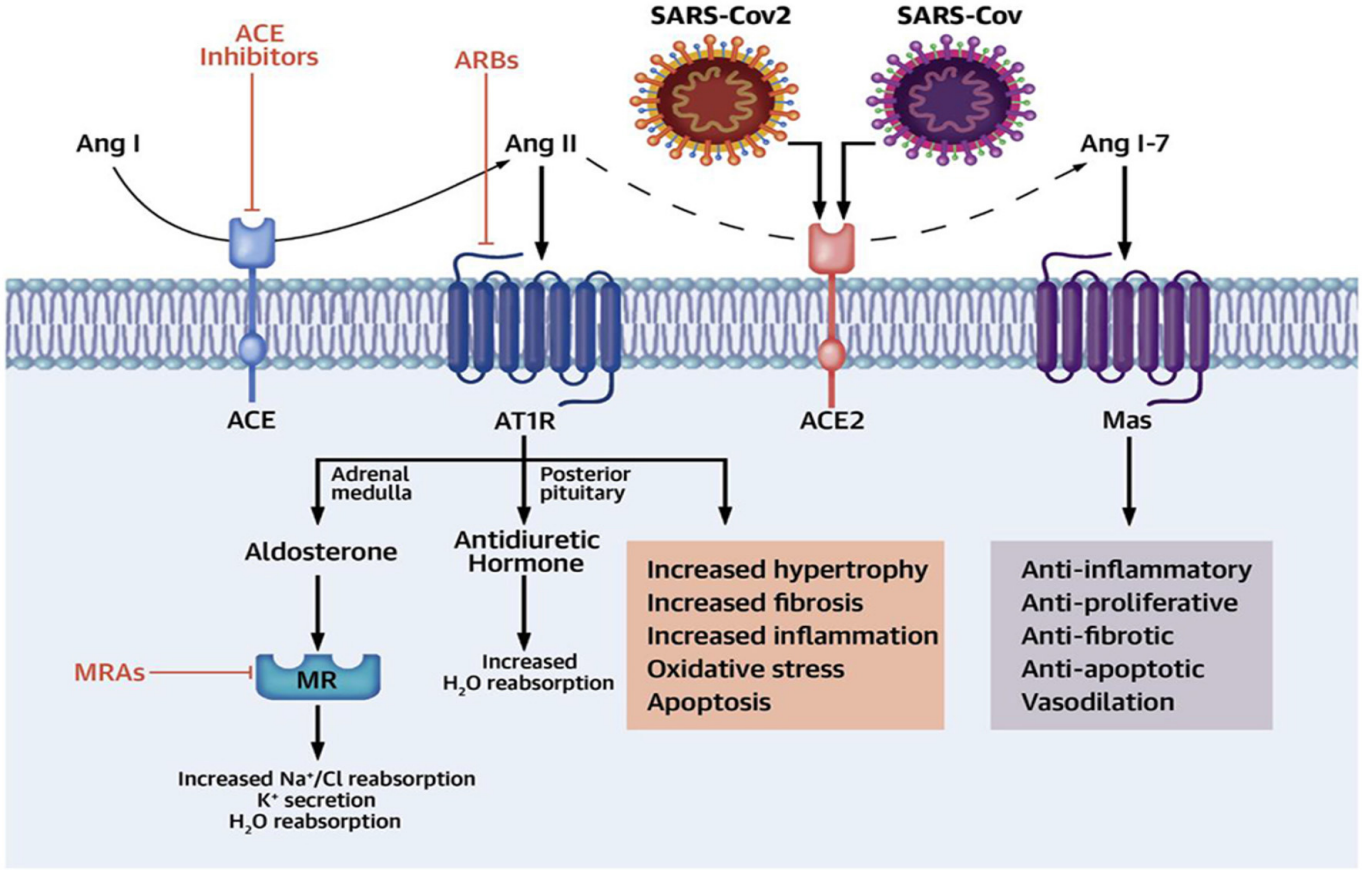
Renin–angiotensin system (RAS) and SARS-CoV-2 infection [adopted from Yehualashet et al. (44)].

The renin–angiotensin system (RAS) is highly deteriorated during SARS-CoV-2 infection, as SARS-CoV-2 spike protein binds to ACE2, leading to a significant downregulation of protective ACE2. ACE2 is involved in the metabolism and conversion of angiotensin I (Ang I) to Ang1–9 and Ang II to Ang1–7. Both Ang1–9 and Ang1–7 have protective effects, exerting anti-inflammatory, anti-apoptotic, and anti-fibrotic effects through activation of Mas receptors (Figure 3) (45). Therefore, reduction and downregulation of ACE2 by SARS-CoV-2 lead to an elevation of vasoconstrictor Ang II causing vasoconstriction, hypertension, and induction of inflammatory and pro-inflammatory activations. A high Ang II concentration is directly linked to ALI and ARDS in Covid-19, so that both angiotensin-converting enzyme inhibitors (ACEIs) and angiotensin receptor blockers (ARBs) are able to reduce the harmful effect of Ang II on the lung through ACE2 upregulation (44).

It has been reported that E2 increases the expression of ACE2 and production of Ang1-7 (46) so it may counteract the deleterious effect of high Ang II-induced-ALI and ARDS. Tao et al. (47) confirmed the association between high Ang II concentration and ALI in mice. Thereby, estrogen therapy may have an important step in the management of Covid-19 patients. Shabbir et al. (39) illustrated that estrogen therapy mitigates endoplasmic reticulum stress (ER) induced by SARS-CoV-2 invasion through activation of cellular unfold protein response and regulation of inositol triphosphate (IP3) and phospholipase C. Baran-Gale et al. (48) reported that E2 inhibits type II transmembrane protease serine (TMPRSS2), necessary for trimming and activation of SARS-CoV-2 spike protein to bind ACE2. Indeed, it has been reported that TMPRSS2 inhibitors, such as camostat and nafamostat, block SARS-CoV-2 entry, mainly in lung cells (49). Different studies have also illustrated that E2 increases the expression of A Disintegrin And Metalloproteinase (ADAMs) mainly ADAM-17, which is involved in cleaving of the ACE2 ectodomain, leading to high-circulating soluble ACE2 that neutralizes SARS-CoV-2 and prevents its binding to the ACE2 membrane (50). Thus, E2 reduces SARS-CoV-2 infectivity through modulation of cellular ACE2/TMPRSS2/ADAM-17 axis expression.

## Estrogen and Signaling Pathway in Covid-19

The Covid-19 severity is linked to the activation of inflammasomes, which are multi-protein complexes that control the activation of pro-inflammatory cytokines through intracellular caspase-1. The nod-like receptor pyrin domain 3 (NLRP3) is the most common inflammasome engaged with immunity against different pathogens (51). Raut et al. (52) illustrated that estrogen therapy inhibits the activation of NLRP3 inflammasomes, so E2 ameliorates airway inflammation and hyper-responsiveness through inhibition of NLRP3 inflammasome-induced proinflammatory cytokine release (53), therefore attenuating the SARS-CoV-2-induced hyper-inflammation and ALI.

Furthermore, protease dipeptidyl peptidase 4 (DPP4) is also regarded as an entry point for SARS-CoV-2 and linked to viral pathogenesis and hyper-inflammation. Besides, DPP4 inhibitors mitigate SARS-CoV-2 infections and reduce the mortality in severe Covid-19 patients (54). It has been shown that E2 by ER-α inhibits DPP4 gene transcription through suppression of NF-kB signaling (55), and for that reason, high E2 levels in women may reduce DPP4 expression in lung and adipose tissues, thereby lessening the infectivity and pathogenicity of SARS-CoV-2.

Moreover, the active site of DPP4 (CD26) contains adenosine deaminase (ADA) which is involved in the metabolism of adenosine to inosine. Adenosine has anti-inflammatory and immunosuppressive effects and can protect against ALI and ARDS development (56). Therefore, ADA inhibitors, such as pentostatin, may have a role in preventing and attenuating SARS-CoV-2 infection and associated hyper-inflammation in the late phase of Covid-19 (57). Mohamadi et al. (58) illustrated that E2 through ER-α activates adenosine receptors, so that estrogen might be of value in repressing ALI and ARDS in the late phase of Covid-19 through modulation of the DPP4/CD26/adenosine signaling pathway.

**Figure 4:**
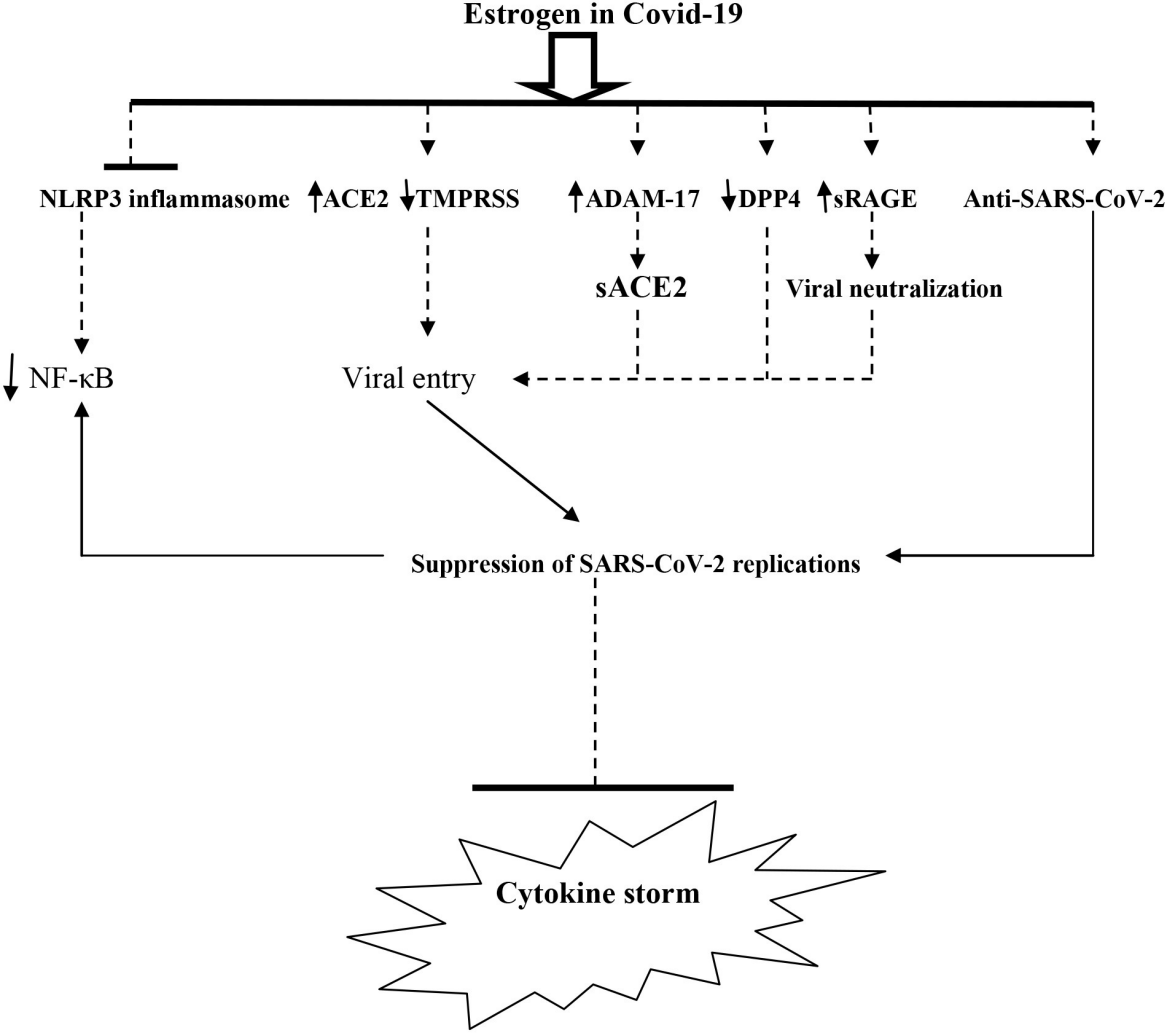
The potential role of estrogen against SARS-CoV-2 pathogenesis and cytokine storm. TMPRRSS, type II transmembrane protease serine; ADAMA-17, A Disintegrin And Metalloproteinase-17; DPP4, Dipeptidyl Peptidase 4; sRAGE, soluble receptor for advanced glycation end-Product; sACE2, soluble angiotensin-converting enzyme 2.

Moreover, the receptor for advanced glycation end products (RAGE) is a member of immunoglobulin superfamily proteins, present in two forms, membrane RAGE (mRAGE) and soluble RAGE (sRAGE). mRAGE has inflammatory effects through activation of NF-kB, while sRAGE has anti-inflammatory effects through upregulation of ACE2 and anti-inflammatory cytokines. The RAGE pathway is mainly expressed in lung tissue and linked to development of acute and chronic lung injuries (59). In Covid-19, SARS-CoV-2 activates mRAGE at pulmonary alveolar cells leading to induction of severe inflammatory reactions (60). Indeed, it has been reported that sRAGE concentration is reduced with aging, which might explain the susceptibility of the elderly to Covid-19. However, in young and asymptomatic Covid-19 patients, the sRAGE concentration is high, while in severe Covid-19, the sRAGE level is significantly reduced, and so a low sRAGE level is linked to the development of ALI and ARDS (61). Finally, it has been shown that estrogen modulates the RAGE pathway through upregulation of sRAGE and reduction of mRAGE (62), so that the administration of E2 in patients with severe Covid-19 may have a lung-protective effect through modulation of the lung RAGE signaling pathway (Figure 4).

From the above, the diverse effects of estrogen on SARS-CoV-2 genome replication and associated immunological response in Covid-19 favor the hypothesis of an interplayed action among SARS-CoV-2 infection, immune response, and estrogen hormone which determine and regulate the gender-dependent final outcomes of Covid-19. Up to date, available data on the precise mechanisms determining the diverse predisposition and outcomes of SARS-CoV-2 infection, either hormonal or immunological, are fragmented and not comprehensive, but the disclosure in order to ascertain gender-specific molecular pathways involved is hopeful. Therefore, emergence of future clinical trials and large-scale prospective studies are warranted in this regard to confirm the supreme importance of estrogen therapy in the management of Covid-19 mainly in postmenopausal women.

## Conclusion

This study illustrates that estrogens have noteworthy anti-inflammatory and immunomodulation effects in Covid-19. Also, estrogen hormone reduces SARS-CoV-2 infectivity through modulation of pro-inflammatory signaling pathways. This study highlighted the potential protective effect of estrogen against Covid-19 and recommended for future clinical trials and prospective studies to elucidate and confirm this protective effect.

## Author Contributions

All authors listed have made a substantial, direct and intellectual contribution to the work, and approved it for publication.

## Conflict of Interest

The authors declare that the research was conducted in the absence of any commercial or financial relationships that could be construed as a potential conflict of interest.
